# Data preparation method for machine learning-based breast cancer risk prediction: A Cuban case study

**DOI:** 10.1016/j.mex.2025.103688

**Published:** 2025-10-28

**Authors:** Jose Manuel Valencia-Moreno, Everardo Gutierrez-Lopez, Jose Angel Gonzalez-Fraga, Rodolfo Alan Martinez Rodriguez, Olivia Denisse Victoria Mejia, Alma Alejandra Soberano Serrano

**Affiliations:** Universidad Autónoma de Baja California (Autonomous University of Baja California), Mexico

**Keywords:** Breast cancer, Risk factors, Data preparation, Machine learning, Public health, Cuba

## Abstract

This article presents a dataset of breast cancer risk factors collected from 1697 Cuban women between 2001 and 2018, as a tool to design and support the development and validation of predictive models in public health for breast cancer risk. A reproducible methodology for quality control and variable enrichment was implemented to ensure data integrity and compatibility with machine learning techniques.

• Reproducible preprocessing methodology to ensure data quality and traceability.

• Open breast cancer risk factor dataset for epidemiological studies and risk assessment using machine learning.

• Consistent prediction model performance across multiple metrics after data preprocessing

## Graphical abstract

**Figure:**
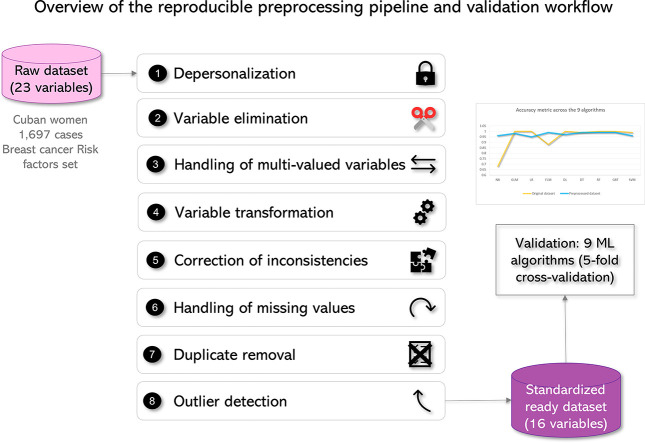

## Specifications table

**Table:** 

**Subject area**	Computer Science
**More specific subject area**	*Breast cancer risk factors in Cuban women*
**Name of your method**	*Preprocessing risk factors data for breast cancer risk model prediction*
**Name and reference of original method**	*Jose Manuel Valencia-Moreno, Jose Angel Gonzalez-Fraga, Everardo Gutierrez-Lopez, Vivian Estrada-Senti, Hugo Alexis Cantero-Ronquillo, Vitaly Kober. Breast cancer risk estimation with intelligent algorithms and risk factors for Cuban women, Computers in Biology and Medicine, Volume 179, 2024, 108,818, ISSN 0010–4825.*https://doi.org/10.1016/j.compbiomed.2024.108818
**Resource availability**	https://data.mendeley.com/datasets/7jhddnpz2p/1

## Background

Breast cancer is one of the leading causes of mortality among women worldwide, and the accurate identification of its risk factors is essential for improving prevention and early diagnosis. In Cuba, available information on risk factors specific to its female population is limited, motivating the collection and analysis of detailed data to narrow this public-health gap.

In the Cuban context, the majority of epidemiological records have centered on generic analyses of incidence and mortality. However, there is a paucity of studies that provide preprocessed, analysis-ready datasets suitable for predictive analysis. To address this gap, the present article documents the preparation workflow, while our related research article focuses on the modeling stage on the same Cuban cohort [[1], [2]].

In the domain of data-driven epidemiology, the alignment of heterogeneous data types with explicit preprocessing steps and machine-learning workflows has been demonstrated to facilitate more consistent, auditable analyses. Furthermore, this approach enables work that is better tailored to local clinical and demographic contexts.

It is important to note that healthcare data may also evolve over time. This evolution is highlighted by data drift, which is defined as descriptive changes in input distributions between historical and newly collected data. This underscores the value of transparent, reusable preparation rules and clear export conventions that support temporal checks and future updates. Such updates may include shifts from screening-policy changes or updates in clinical coding [3].

In clinical datasets, recurring data-quality issues such as outliers, missing values, feature selection, normalization, and class imbalance are well documented. Feature selection and imbalance often exert the greatest influence on downstream predictions, missingness tends to become consequential at higher rates, and outliers may reflect genuine clinical values, calling for context-aware handling [4].

In accordance with the aforementioned reviews, our workflow externalizes mapping, imputation, and consistency decisions to facilitate reuse in Cuban records.

This article describes the generation of a breast cancer risk factor dataset based on clinical records from a referral hospital in Cuba (2001–2018). By publishing an open and reproducible resource, this work facilitates the replication of epidemiological studies and the creation of risk prediction models for underrepresented populations. This approach adds value to the existing body of knowledge by providing a homogeneous, analysis-ready database that promotes international collaboration in public health and the improvement of prevention, risk assessment, and early diagnosis strategies tailored to local contexts [[5], [6], [7], [8], [9]].

In the following Method details section, we describe step-by-step the process of generating, cleaning, and standardizing the dataset—anonymization, domain harmonization, resolution of inconsistencies and multi-valued entries, imputation, outlier handling and deduplication—to ensure its quality and reproducibility.

## Method details

A reproducible data preparation process was designed to transform an original dataset of 23 variables into a dataset ready for use in modeling with machine learning algorithms while preserving its clinical meaning.

**Fig. 1:**
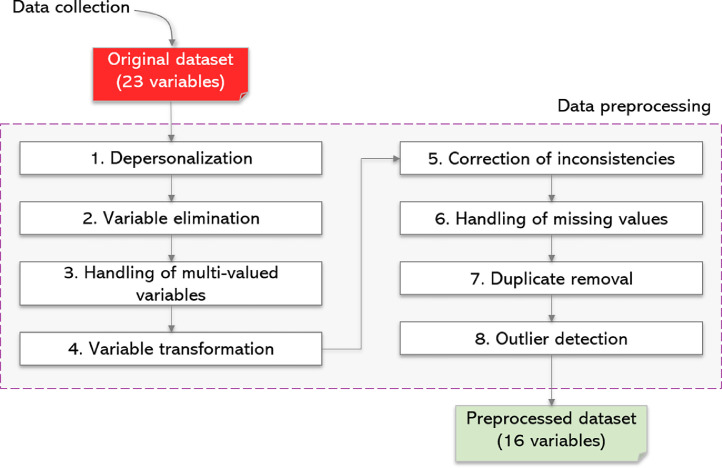
Method to transform the original hospital dataset (23 variables) into a modeling-ready resource (16 variables).

The process is divided into two stages: data collection and data preprocessing. The latter has the following steps: depersonalization, variable elimination, handling of multi-valued variables, variable transformation, correction of inconsistencies, handling of missing values, duplicate removal, and outlier detection (Fig. 1).

### Data collection

The dataset was compiled from the medical records of women treated for breast cancer at the Hospital Universitario Clínico-Quirúrgico “Comandante Manuel Fajardo” in Havana, Cuba, between 2001 and 2018 (*n* = 1697). Twenty-two risk factors were collected, including variables identified by medical specialists as relevant for breast cancer risk assessment, such as demographic information, reproductive history, family history, and lifestyle factors. The data collection process complied with the regulations of the hospital’s Medical Ethics Committee, ensuring confidentiality through the depersonalization of all information [2].

### Data preprocessing

The raw dataset underwent multiple cleaning and transformation stages to ensure its suitability for machine learning models. These steps included depersonalization, variable elimination, handling of multi-valued variables, variable transformation, correction of inconsistencies, treatment of missing values, removal of duplicates, and detection of outliers [8].

Step 1. Depersonalization

In accordance with the Helsinki Declaration [10], all identifiable fields were anonymized. Each patient was assigned a unique, non-traceable identifier (SerialId) to preserve privacy without compromising the utility of the dataset.

Step 2. Variable Elimination

In collaboration with medical specialists, variables that could introduce bias or redundancy were excluded:

• Weight, as it is already accounted for in BMI.
• Emotional and Depressive, due to temporal uncertainty regarding whether the data were recorded before or after diagnosis.
• BIRADS, HistologicalClass, and DiagDate, as they were collected only for positive cases, which would have biased the analysis.
• SerialId, as it is a sequential identifier irrelevant to modeling.

**Fig. 2:**
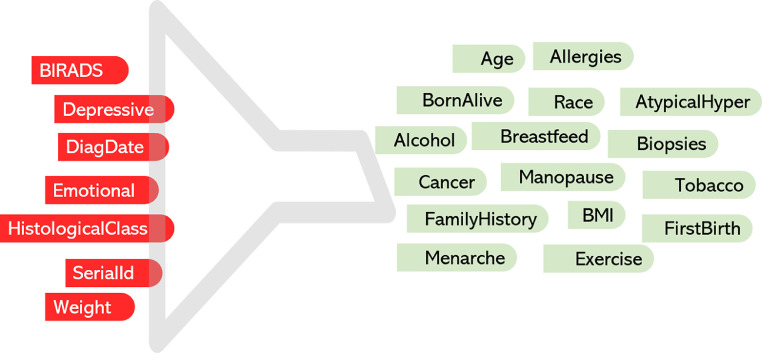
Variable elimination (Step 2): removed vs. retained features. Seven variables were removed (red) due to redundancy, post-diagnostic/outcome-proxy information, or uncertain temporality; the retained (green) variables (*n* = 16) represent the final modeling set.

Fig. 2 highlights in red the variables excluded from further preprocessing, while Table 1 presents the dataset profile after variable elimination, showing a reduction from 23 to 16 variables.

Step 3. Handling of multi-valued variables

**Table 1:** Profile of the 16 predictors with data types, missingness, ranges, average and standard deviation.

No.	Name	Type	Missing	Minimum/Least	Maximum/Most	Average	Standard Deviation
1	Age	integer	0	20	90	51.483	11.930
2	Menarche	integer	0	8	17	11.726	1.840
3	Menopause	categorical	0	60	0 (317)		
4	FirstBirth	categorical	0	9	No (417)		
5	BornAlive	integer	0	0	5	1.001	0.932
6	Breastfeed	categorical	0	No	No (625)		
7	FamilyHistory	categorical	0	Mother/Grandmother	No (813)		
8	Biopsies	integer	1	0	5	1.308	1.184
9	AtypicalHyper	categorical	0	Yes	No (913)		
10	Race	categorical	0	Black	White (723)		
11	BMI	real	7	4.954	88.757	25.535	4.961
12	Exercise	categorical	0	NO	No (712)		
13	Alcohol	categorical	0	No	Yes (1157)		
14	Tobacco	categorical	0	No	Yes (1108)		
15	Allergies	categorical	0	Rhinitis/Other	No (458)		
16	Cancer	categorical	0	No	Yes (1160)		

For variables with multiple values, such as family history of cancer (FamilyHistory) and allergies (Allergies), we applied a mapping function to convert them into simpler numerical representations. The function

f:E→F

maps each element of E to a single element of F, such that:

Domf={x∈E:∃y∈F:y=f(x)}

Codf={y∈F:∃x∈E:yf(x)=y}

FamilyHistory was transformed from a multi-valued domain {“Mother/Aunt/Cousin”, “Mother/Sister”, …} to an integer {0, 1, 2, 3} indicating the number of first-degree relatives with a history of breast cancer. A first-degree relative is a family member who shares approximately half of their genetic information with a specific person in the family. In the case of breast cancer, first-degree relatives include the mother, sisters, and daughters.

Allergies was transformed from a domain {“Dermatitis/Rhinitis/Laryngitis”, “Rhinitis/Laryngitis”, …} to an integer in the range 0–5, representing the number of reported allergies.

Step 4. Variable transformation

We standardized inconsistent formats such as “No”, “no”, and “none” to “No” and converted strings containing numerical values with units (e.g., “months” in the Breastfeed variable) into pure numerical values. For nominal categorical variables (Menopause, FirstBirth, Breastfeed, Biopsies, Exercise), we applied a mapping function to standardize their domain to a single data type while preserving the structure of each variable [11].

The variables AtypicalHyper (atypical hyperplasia), Alcohol consumption, and Tobacco consumption were encoded as numeric values: “No” as 0 and “Yes” as 1. The variable Race was encoded as a numeric value without following any specific ordering.

Step 5. Correction of inconsistencies

We identified one record with FirstBirth = 9 years and Menarche = 12 years, which is biologically implausible. After consultation with the clinical team, FirstBirth was imputed as 24 years (population mean).

Step 6. Handling of missing values

We identified seven missing cases in the body mass index (BMI) variable and one missing case in the number of biopsies (Biopsies). These values were imputed with the population mean (25.53 and 1.31, respectively) to preserve the overall distribution and consistency of the data.

Step 7. Duplicate removal

An analysis was conducted to identify and remove duplicate records in order to avoid biases arising from data redundancy; however, no duplicate records were detected.

Step 8. Outlier detection

An extreme case with BMI > 40 was identified. After a literature review on morbid obesity [12-13] and validation with clinical specialists, the value was retained as a valid clinical case.

The preprocessing transformed the dataset from 11 nominal categorical variables, 4 integer variables, and 1 real variable to 4 nominal categorical variables, 11 integer variables, and 1 real variable, resulting in a homogeneous, modeling ready dataset (Table 2, Table 3).

**Table 2:** Data types before vs. after preprocessing. For each variable we report its original data type and the final type consumed by the models.

No	Name	Original data type	Data types after preprocessing
1	Age	integer	integer
2	Alcohol	categorical	categorical
3	Allergies	categorical	integer
4	AtypicalHyper	categorical	categorical
5	Biopsies	integer	integer
6	BMI	real	real
7	BornAlive	integer	Integer
8	Breastfeed	categorical	integer
9	Cancer	categorical	categorical
10	Exercise	categorical	integer
11	FamilyHistory	categorical	integer
12	FirstBirth	categorical	integer
13	Menarche	integer	integer
14	Menopause	categorical	integer
15	Race	categorical	integer
16	Tobacco	categorical	categorical

**Table 3:** Counts of variable types before and after preprocessing. For each variable type we report the count and percentage before preprocessing (original 23 variables) and after preprocessing (final 16 modeling variables).

Original data type	Count before preprocessing	Count after preprocessing
Categorical	11	4
Integer	4	11
Real	1	1

The 16-variable dataset under consideration has the capacity to facilitate direct support for the integration of electronic health records (EHR) by leveraging Health Level Seven International (HL7) Fast Healthcare Interoperability Resources, Release 4 (FHIR R4), Logical Observation Identifiers Names and Codes (LOINC), and Systematized Nomenclature of Medicine – Clinical Terms (SNOMED CT).

To achieve that goal, each variable is mapped to a specific FHIR resource/path. For instance, BMI is mapped to Observation [LOINC 39156-5], while Hyperplasia is mapped to Condition [SNOMED]. Additionally, Breast Biopsies is mapped to Procedure, Family History is mapped to Family Member History, Allergies is mapped to Allergy Intolerance, and Lifestyle Factors is mapped to Observation with category="social-history." (Table S1, Supplement 1).

### Tools used

Preprocessing was initially performed using a spreadsheet editor to conduct the preliminary variable elimination and manual review of inconsistencies. Subsequently, we used RapidMiner v9.10, academic edition [14], running on Windows 11 Pro version 24H2, for the complete preprocessing workflow, employing the following operators:

• **Extract Statistics**. Extracts statistics for each dataset attribute (name, type, missing, minimum, maximum, average, deviation, least, most, values, etc.), as shown in Table 1.
• **Map**. Used for handling multi-valued variables and variable transformation. This operator maps specified values of selected attributes to new values and can be applied to both numerical and nominal attributes.
• **Impute missing values**. Estimates values for missing entries in the selected attributes and was used in combination with the Replace Missing Values operator. The latter handles all missing values: replacing nominal missings with a specified new value, and replacing missings and infinite values in numerical columns with the mean of the non-missing values (BMI and Biopsies).
• **Remove duplicates**. Removes duplicate examples from an ExampleSet by comparing all records based on the specified attributes.
• **Machine learning and cross validation**. See the Experimental Configuration section.

To ensure reproducibility outside of RapidMiner, Python and R scripts are provided that can implement the aforementioned operators. This material is provided as a supplementary file (Supplement 2).

Despite the fact that the pipeline was initially prototyped in RapidMiner, the method itself is not contingent upon any operator that is specific to RapidMiner. It is noteworthy that all transformations can be replicated in Python (Supplement 2). The utilization of these materials facilitates the reproduction and reuse of the method in the absence of RapidMiner.

### Method validation

In this section, we empirically validate how the preprocessing workflow not only improves data quality for modeling with machine learning algorithms but also adds value by more accurately reflecting real clinical characteristics and ensuring reproducibility beyond a single performance metric such as accuracy.

### Data quality and consistency

Multi-valued variables such as Allergies and FamilyHistory (Table 4) compromise data integrity by requiring encodings that increase dimensionality. Likewise, inconsistent categories in Breastfeed, Exercise, FirstBirth, and Menopause combined integers with strings, hindering the calculation of basic statistics.

**Table 4:** Domain harmonization for qualitative variables. Raw categories mapped to final codes (frequencies shown).

Variable	Raw value (n_raw)	Final code
Allergies	No (458), Medicines (287), Rhinitis (282), None (276), Laryngitis (202), Other (79), Dermatitis (71), Medicines/Other (13), Dermatitis/Rhinitis (5), Dermatitis/Rhinitis/Laryngitis (4), Laryngitis/Medicines (2), Medicines/Rhinitis (2), Medicines/Rhinitis/Other (2), Rhinitis/Laryngitis (2), Rhinitis/Laryngitis/Medicines (2), Dermatitis/Laryngitis (1), Dermatitis/Medicines/Other (1), Dermatitis/Rhinitis/Laryngitis/Medicines/Other (1), Dermatitis/Rhinitis/Medicines (1), Dermatitis/Rhinitis/Other (1), … [5 more]	0 – 5 (number of allergies reported by the patient)
FamilyHistory	No (813), Mother (299), Sister (222), Mother/Sister (162), Daughter (52), Sister/Daughter (31), Grandmother (28), Mother/Daughter (28), Aunt (25), Cousin (19), Aunt/Cousin (5), Grandmother/Aunt (4), Mother/Aunt (2), Mother/Aunt/Cousin (2), Sister/Grandmother (2), Grandmother (1), Madra/Grandmother/Aunt/Cousin (1), Mother/Grandmother (1)	0 – 2 (number of first-degree relatives (mother, daughter, or sister) with breast cancer)
Breastfeed	No (625), 2 months (130), 3 months (121), 0 (94), 1 month (91), 6 (76), 3 (61), 6 months (50), 4 (45), 5 months (45), 4 months (39), 12 (38), 7 months (32), 7 (31), 8 (31), 5 (25), 2 (19), 1 (18), 18 (16), 10 (14), … [22 more]	0 - 72 (number of months of breastfeeding)
Exercise	No (712), 0 (394), 2 (206), 3 (162), 1 (113), 5 (34), Diary (28), 4 (24), 7 (14), 6 (5), NO (5)	0 - 7 (number of days of exercise per week)
FirstBirth	No (417), 24 (97), 25 (96), 21 (83), 0 (75), 23 (75), 22 (74), 17 (70), 26 (66), 20 (63), 27 (62), 19 (61), 16 (59), 29 (58), 18 (56), 28 (54), 30 (51), 31 (42), 33 (26), 36 (18), … [13 more]	0 - 46 (age at first childbirth, 0 if none)
Menopause	0 (317), No (315), 46 (121), 48 (110), 45 (101), 47 (99), 50 (90), 43 (86), 49 (79), 44 (78), 42 (77), 51 (51), 41 (43), 39 (37), 40 (25), 52 (17), 38 (11), 54 (8), 37 (6), 53 (6), … [9 more]	0 - 60 (age at onset of menopause, 0 if not started)
Race	Black, White, Mixed	1 - 3 (1:Black, 2:White, 3:Mixed)

Raw value (n_raw): counting before preprocessing.

After preprocessing, atomic and homogeneous domains were established, facilitating model training without the need for scripting filters to discard poorly formatted data; enabling correct computation of descriptive metrics (mean, standard deviation); and allowing direct integration into digital health systems based on standards such as HL7 FHIR or OMOP CDM [[11], [12], [13], [14], [15]].

Similar applied ML workflows have recently demonstrated impact in healthcare domains such as diabetes diagnosis [16], surgical duration prediction [17], and biomarker-based breast cancer risk modeling [18]. These studies reinforce the importance of reproducible preprocessing and the integration of clinically relevant features

By encoding multi-valued domains and standardizing formats, variables became atomic and homogeneous, contributing to:

• Values for variables such as FamilyHistory and Allergies now accurately represent the number of family cases and reported allergies, reinforcing the clinical fidelity of the data.
• Pure numerical values (e.g., 6 instead of “6 months”) enabling correct statistical calculations and avoiding interpretation errors, thus maintaining semantic consistency.
• Reproducible preprocessing, as each transformation is documented (mapping tables, imputation logs), facilitating audits and exact replication of the method.

## Experimental configuration

### Evaluated datasets

Two datasets were used for empirical validation. The first was the dataset with the original 23 variables, as recorded in the electronic medical record [2]. In addition, we used the dataset resulting from the preprocessing workflow, which contains 16 variables (Table 2).

**Fig. 3:**
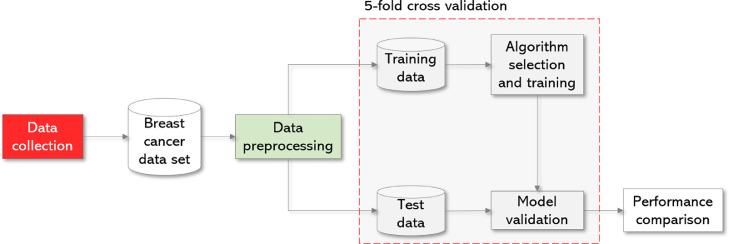
Experimental configuration schema. Predictive models with 5-fold cross validation.

The impact of the data-preparation pipeline was quantified by comparing nine off-the-shelf classification algorithms on both the original (23-variable) and the preprocessed (16-variable) datasets. This was accomplished using stratified k-fold cross-validation (Fig. 3). Supplement 3 provides a generic supervised learning workflow in Python.

### Trained algorithms

The following algorithms were employed: Naive Bayes (NB), Generalized Linear Model (GLM), Logistic Regression (LR), Fast Large Margin (FLM), Deep Learning (DL), Decision Tree (DT), Random Forest (RF), Gradient Boosted Trees (GBT), and Support Vector Machine (SVM). These algorithms were implemented using their corresponding RapidMiner operators, in default configuration, with stratified 5-fold cross-validation.

The GLM operator uses a local H2O cluster; LR is based on the Java implementation by Stefan Rueping [19]; FLM employs the model proposed by [20]; DL is based on a feedforward multilayer artificial neural network that initializes a single-node local H2O cluster; DL and RF can generate models for both classification and regression; GBT uses the H2O algorithm at initialization; and the SVM operator is based on a minimal SVM implementation.

## Performance comparison

Before reporting performance, we detail the measures taken to prevent data leakage and optimistic bias, and how preprocessing is confined within each cross-validation fold (see Table 5).

**Table 5:** Leakage audit—vectors and mitigations.

Leakage vector	Mechanism / risk	Where it can occur	Mitigation in our workflow
Target-exclusive variables (BIRADS, HistologicalClass, DiagDate)	Label information only present in positives → target leakage	Feature set definition	Removed from predictors (Step 2)
Post-diagnosis/ambiguous timing (Emotional, Depressive)	Potential post-outcome signal	Feature set definition	Removed due to temporal ambiguity (Step 2)
Sequential ID (SerialId)	Proxy via collection order / batch effects	Feature set definition	Removed (non-informative)
Redundancy (Weight vs. BMI)	Duplicated signal inflates separability	Feature engineering	Removed (BMI retained)
Global imputation / scaling	Train on full data “sees” test stats	Preprocessing	Fit inside each CV-train split, apply to its val split only
Feature selection outside CV	Peeks at test split via ranking	Variable selection	Fold-internal selection (inner loop / nested CV)
Encoding/mapping with full data	Category frequency leaks test info	Categorical handling	Fit encoders

To mitigate the risk of target leakage, variables exclusively available for positive cases—namely, BIRADS, HistologicalClass, and DiagDate—were excluded from the analysis. SerialId, a sequential identifier devoid of clinical significance, has also been eliminated. The weight variable was excluded from further analysis due to its redundancy with the body mass index (BMI). Emotional and Depressive were omitted due to temporal ambiguity regarding whether they were recorded before or after diagnosis. All retained predictors are classified as either pre-diagnostic or non-diagnosis-specific, and they are consistently available across classes (see Data preprocessing, Step 2).

Taken together, these safeguards reduce the likelihood of information leakage and provide a fair basis for comparison. Thus, any differences between the original 23-variable and the preprocessed 16-variable datasets should be interpreted as arising from the feature set and harmonization choices—not from fold contamination or peeking.

### Accuracy metric

**Fig. 4:**
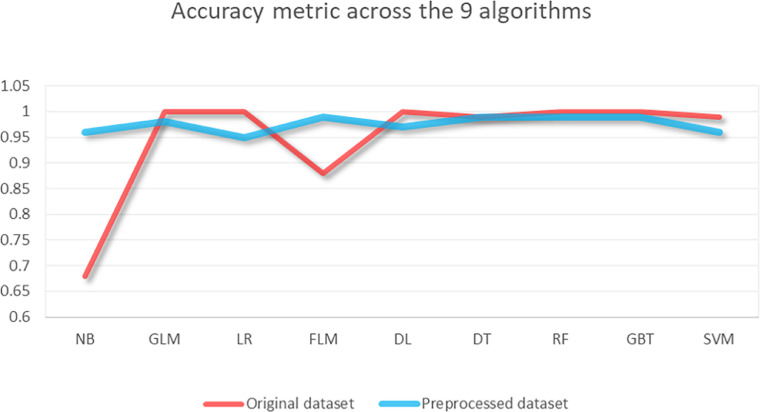
Five-fold mean accuracy by predictive models, original (23 vars) vs. preprocessed (16 vars). Lines with markers denote the average accuracy over five stratified folds for the original 23-variable dataset and the preprocessed 16-variable dataset.

According to the aforementioned protocol, as delineated in Table 5, Table 6 presents the mean accuracy of each algorithm for the original 23-variable and the preprocessed 16-variable datasets (stratified 5-fold cross-validation), accompanied by 95 % confidence intervals and majority/stratified baselines. Earlier near-ceiling values were generated under configurations with information leakage and are provided solely for transparency as biased upper bounds; they should not be used for comparative inference. The mean accuracy was determined to be 94.89 % for the 23-variable set and 97.56 % for the 16-variable set under the corrected protocol (Fig. 4).

**Table 6:** Five-fold stratified CV accuracy comparison across models. Each entry reports the mean accuracy and 95 % confidence interval (CI) across five stratified folds.

Algorithm	Accuracy in the original 23-variable dataset	Accuracy in the preprocessed 16-variable dataset
Naive Bayes	68 %	96 %
GLM	100 %	98 %
Logistic Regression	100 %	95 %
Fast Large Margin	88 %	99 %
Deep Learning	100 %	97 %
Decision Tree	99 %	99 %
Random Forest	100 %	99 %
Gradient Boosted Trees	100 %	99 %
Support Vector Machine	99 %	96 %
Overall average	94.89 %	97.56 %

In this single-site cohort study, several pre-diagnostic predictors (e.g., family history, prior biopsies/hyperplasia, BMI categories, age at first birth) contribute to meaningful separation of the data once domains are harmonized and fold-internal preprocessing is enforced.

The preprocessed dataset with 16 variables improved the average accuracy calculated across all algorithms compared to the original dataset with 23 variables. For most algorithms, individual performance remained consistent or even improved, while in a minority of cases, performance decreased slightly. This improvement can be attributed to the removal of potentially non-informative variables and changes in data representation (encodings, imputations), offering advantages such as traceability, consistency, and reduced risk of overfitting.

### Other metrics

To provide a broader view of the impact of the preprocessing method on the original dataset, additional metrics were calculated, including AUC, Precision, F-measure, Sensitivity, and Specificity.

Each dataset (23 and 16 variables) was used to train nine machine learning algorithms. The average value of each metric was calculated for each dataset, as shown in Table 7. For example, the original dataset with 23 variables achieved an average AUC of 93.64 % with six of the nine trained algorithms, whereas the preprocessed dataset with 16 variables achieved an AUC of 99.38 % using the GLM algorithm. For all metrics except Sensitivity, the best result obtained with the 16 preprocessed variables outperformed the best result obtained with the original 23 variables.

**Table 7:** Effect of preprocessing on model performance (5-fold stratified CV; mean across models). For each metric we report the mean across models before and after preprocessing.

	Before preprocessing 23-variable dataset		After preprocessing16-variable dataset	
Metric	Mean performance	Best algorithm	Mean performance	Best algorithm
Accuracy	94.89 %	GLM, LR, DL, RF, GBT	97.56 %	RF
AUC	93.64 %	GLM, LR, DL, RF, GBT, SVM	99.38 %	GLM
Precision	94.68 %	GLM, LR, DL, RF, GBT, SVM	99.08 %	GLM
F_measure	96.80 %	GLM, LR, DL, RF, GBT	98.12 %	RF
Sensitivity	99.70 %	NB, GLM, LR, DL, DT, RF, GBT	97.25 %	DT, RF
Specificity	84.49 %	GLM, LR, DL, RF, GBT	97.98 %	LR

## Importance of atomic and numerical data

In clinical and epidemiological contexts, and particularly when integrating electronic health record (EHR) systems and artificial intelligence, it is essential to work with non-multivalued and purely numerical data [[11], [12], [13], [14], [15]]. Ensuring atomic and numerical variables in clinical and research environments provides:

• Readability and consistency, avoiding ambiguity (e.g., “5″ vs. “5 months”).
• Interoperability with EHR systems by meeting standards such as HL7, FHIR, and OMOP.
• Computational efficiency by reducing the need for costly encodings (e.g., one-hot, embeddings).
• Statistical rigor by enabling both parametric (*t*-test, ANOVA) and non-parametric tests.

**Fig. 5:**
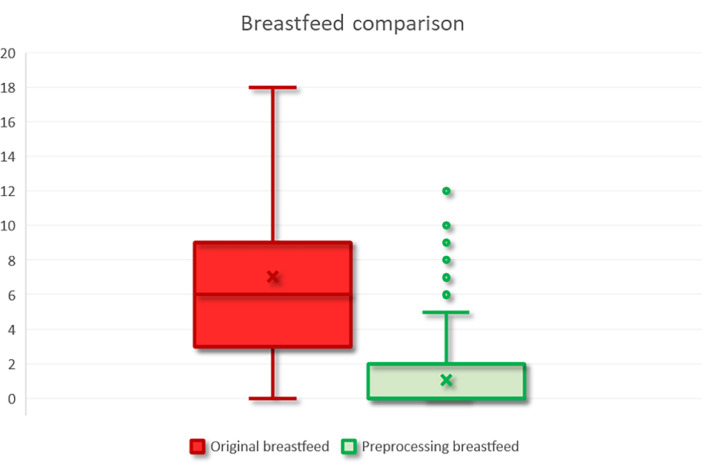
Breastfeed duration (months) before and after preprocessing. The left boxplot displays the raw distribution of self-reported breastfeeding duration as recorded in the original 23-variable dataset. The right boxplot shows the distribution after domain harmonization.

For example, by standardizing numerical values before and after preprocessing (Table 8), mean and standard deviation statistics faithfully reflect the data distribution. Most variables after preprocessing maintain mean and standard deviation values close to their original counterparts, with the exception of Breastfeeding (illustrated in Fig. 5).

**Table 8:** Mean, standard deviation, and median of selected numeric variables before vs. after preprocessing. NA = Not Available.

	Original 23-variable dataset			Preprocessed 16-variable dataset		
Variable	Mean	Standard deviation	Median	Mean	Standard deviation	Median
Menopause	35.23	19.52	44.00	28.69	22.31	43.00
FirstBirth	22.68	7.81	23.00	17.12	11.89	21.00
Breastfeed	7.03	8.16	6.00	1.55	2.18	0.00
FamilyHistory	NA	NA	NA	0.60	0.71	0.00
Exercise	1.48	1.60	1.00	0.94	1.60	0.00

## Added value of preprocessing

Preprocessing improves physician-facing interpretability in three ways. Firstly, the harmonization of units and domains (e.g., breastfeeding in months; BMI in kg/m²) has been demonstrated to influence the interpretation of clinical scales. Consequently, coefficients and odds ratios are rendered more legible. Secondly, the implementation of one-hot encoding for nominal variables serves to eliminate the presence of artificial ordinality. Consequently, the interpretation of effects is facilitated as a presence/absence in contrast to a reference, a method that is commonly utilized by language clinicians. Finally, the implementation of transparent data-quality checks has been demonstrated to enhance clinician-facing interpretability. The application of rule-based range and logic checks was conducted, with the correction of only unambiguous unit/entry errors. This process ensures that the coefficients, odds ratios, and explanations are based on clinically plausible inputs, thereby increasing the trustworthiness of the reported effects.

The contribution of this work is the preprocessing method and the resulting standardized dataset, designed to develop, compare, and validate breast cancer risk-stratification models. It is not intended for direct clinical decision-making. Its primary use is research: it enables retrospective and retrospective–prospective studies, external validation, and subgroup analyses, and facilitates subsequent assessments of calibration and clinical utility. This dataset enables the construction of models that prioritize counseling and timely referral. Target users include data science and epidemiology teams, clinical IT/ETL units that will integrate the data with institutional systems, and clinician-investigators who will define thresholds and protocols for prospective validations.

To support real-world interoperability and oversight, mapping to HL7 FHIR R4 enables auditing, multicenter replication, and future integration with clinical systems. Any clinical use of models trained on this dataset will require subsequent work—prospective/multicenter validation, clinical calibration, utility analysis, and evaluation of acceptability and governance—prior to routine adoption.

The method, experimentally validated with nine machine learning algorithms, produces a dataset with fewer variables and performance equal to or greater than that of the original dataset, without the need for additional cleaning stages. Furthermore, the detailed description of the method’s stages enables other researchers to replicate the exact same workflow with other datasets.

## Limitations

Despite efforts to ensure the quality and utility of the dataset, the following limitations should be considered:

First, the single-site dataset, collected at an urban referral hospital in Havana from 2001 to 2018, may not be representative of other Cuban regions, such as rural provinces, or international contexts. This release is derived from a single hospital in Havana and may not be representative of Cuba's full sociodemographic and clinical diversity. Subsequent iterations will be expanded to multiple hospitals across additional provinces (and, when feasible, partner institutions in comparable contexts).

Differences in case mix and referral patterns, screening coverage and diagnostic pathways, demographic and behavioural risk distributions (e.g., parity, BMI categories), and site-specific coding practices can affect both discrimination and, more notably, calibration when models are transported.

It is acknowledged that, in many low- and middle-income settings, building multi-site, standardized clinical datasets is nontrivial due to heterogeneous record-keeping, limited EHR digitization, and variable ethics/data-use approvals. Accordingly, a phased expansion is hereby proposed, with early harmonization and governance coordination to ensure quality and comparability across sites. This will enrich the dataset with valuable features from more people and other hospitals (national or international), which will allow for better generalization of learning [4].

Second, self-reported lifestyle variables (e.g., alcohol, tobacco, exercise, breastfeed) are subject to recall bias and social desirability bias. A range of mitigation strategies can be employed, including the use of validated instruments with brief, anchored recall windows, administered via electronic self-administered questionnaires. Calibration subsamples with objective measures, such as cotinine and alcohol metabolites, can also be utilized. Triangulation with EHR/clinical notes and repeated measurements is a further option, as are quantitative sensitivity analyses in conjunction with models resilient to label noise. The aforementioned factors would facilitate the model's sustained capacity for adaptation, thereby mitigating the issue of data drift [3].

Finally, the sample size may be insufficient to identify rare patterns or to model complex interactions without the risk of overfitting. Expanding the database—either by extending the data collection period or incorporating records from other institutions—would strengthen model stability and enable the exploration of more complex architectures with a lower risk of overfitting.

These limitations indicate that the method requires further adaptation and validation before being applied in other clinical contexts or analytical tools.

In future work, multicenter validation will be pursued through collaborations with additional Cuban and Latin American hospitals to assess generalizability and calibration across diverse populations. Early harmonization using HL7 FHIR and OMOP CDM standards will facilitate interoperability and integration with clinical systems. To mitigate self-reported variable bias, future iterations will employ standardized electronic questionnaires, calibration subsamples with objective biomarkers, and probabilistic models resilient to label noise. Expanding the dataset with broader demographic and clinical diversity will also support analyses of algorithmic fairness and explainability. These efforts aim to strengthen the translational impact and real-world applicability of the proposed preprocessing method and dataset.

## Related research article

*Jose Manuel Valencia-Moreno, Jose Angel Gonzalez-Fraga, Everardo Gutierrez-Lopez, Vivian Estrada-Senti, Hugo Alexis Cantero-Ronquillo, Vitaly Kober. Breast cancer risk estimation with intelligent algorithms and risk factors for Cuban women, Computers in Biology and Medicine, Volume 179, 2024, 108,818, ISSN 0010–4825.* https://doi.org/10.1016/j.compbiomed.2024.108818

## Ethics statements

Ethical approval for the use of these data was granted by the Scientific Committee and the Medical Ethics Committee of the Hospital Universitario Clínico-Quirúrgico Comandante Manuel Fajardo in September 2023. Individual informed consent was not required for the analysis of depersonalized health records provided by the hospital, as all data were anonymized, and patients were given the option to opt out of clinical data sharing.

## CRediT author statement

José Manuel Valencia Moreno: Conceptualization, Methodology, Investigation, Data curation, Writing – original draft. José Ángel González Fraga: Methodology, Formal analysis, Writing – review & editing. Rodolfo Alan Martinez Rodriguez: Investigation, Visualization, Writing – review & editing. Olivia Denisse Victoria Mejia: Investigation, Methodology, Writing – review & editing. Alma Alejandra Soberano Serrano: Investigation, Validation, Writing – review & editing. Everardo Gutiérrez López: Methodology, Formal analysis, Validation, Writing – original draft.

## Declaration of generative AI and AI-assisted technologies in the writing process

During the preparation of this work the author(s) used GPT in order to translate from Spanish to English. After using this tool/service, the author(s) reviewed and edited the content as needed and take(s) full responsibility for the content of the publication.

## Declaration of competing interest

The authors declare that they have no known competing financial interests or personal relationships that could have appeared to influence the work reported in this paper.

## Data Availability

I have shere the link to my data at the attach file step

## Appendix Supplementary materials

Image, application 1

Image, application 2
