# Supplementary material for: Data preparation method for machine learning-based breast cancer risk prediction: A Cuban case study
Source: MethodsX. 2025 Oct 28;15:103688. doi: 10.1016/j.mex.2025.103688 (PMC12637264; doi:10.1016/j.mex.2025.103688)
Supplement: Supplementary file 1 [file mmc1.docx]

<https://data.mendeley.com/datasets/7jhddnpz2p/1>
