# Supplementary material for: Data preparation method for machine learning-based breast cancer risk prediction: A Cuban case study
Source: MethodsX. 2025 Oct 28;15:103688. doi: 10.1016/j.mex.2025.103688 (PMC12637264; doi:10.1016/j.mex.2025.103688)
Supplement: Supplementary file 2 [file mmc2.docx]

**Supplementary material**

**Data preparation method for machine learning-based breast cancer risk prediction: A Cuban case study**

Jose Manuel Valencia-Moreno, Everardo Gutierrez-Lopez, Jose Angel Gonzalez-Fraga, Rodolfo Alan Martinez Rodriguez, Olivia Denisse Victoria Mejia, Alma Alejandra Soberano Serrano.

Universidad Autónoma de Baja California (Autonomous University of Baja California)

**Ref.: MEX-D-25-01519**

**Supplement 1.**

**FHIR integration highlights.**

**Variables map to standard resources/paths:**

- Demographics → Patient (e.g., birthDate used to derive age).
- Lifestyle & vitals → Observation (category: social-history/vital-signs), e.g., BMI (LOINC 39156-5), tobacco/alcohol use (LOINC/SNOMED per local ValueSets).
- Breast biopsies → Procedure (coded with SNOMED; link to Condition if present).
- Hyperplasia (histologic) → Condition (SNOMED).
- Family history of breast cancer → FamilyMemberHistory (condition.code: SNOMED).
- Allergies → AllergyIntolerance.
- Race/ethnicity → country/profile-specific extension (or local Extension) as applicable.

**Table S1.**

Mapping variables to FHIR resources/ path.

| **Variable** | **FHIR Resource** | **Element / Notes** | **Terminology (suggested)** |
| --- | --- | --- | --- |
| Age | Patient | birthDate (derive age at index) | - |
| Alcohol | Observation | category=social-history; ValueSet local/standard | LOINC/SNOMED per profile |
| Allergies | AllergyIntolerance | code | SNOMED CT / local |
| AtypicalHyper | Condition | code = hyperplasia concept | SNOMED CT |
| Biopsies | Procedure | code = breast biopsy | SNOMED CT |
| BMI (kg/m²) | Observation | valueQuantity; category=vital-signs | **LOINC 39156-5** |
| BornAlive | Observation | valueInteger | local code + binding |
| Breastfeed | Observation | valueQuantity; use dataAbsentReason=not-applicable for nulliparous | local code + binding |
| Exercise | Observation | valueQuantity | LOINC/SNOMED (per local ValueSet) |
| FamilyHistory | FamilyMemberHistory | condition.code = breast cancer (SNOMED) | SNOMED CT |
| FirstBirth | Observation | valueQuantity | local code + binding |
| Menarche | Observation | valueQuantity | local code + binding |
| Menopause | Observation | valueQuantity or boolean; use dataAbsentReason when not applicable | local code + binding |
| Race | Patient (+ Extension) | Country/profile-specific extension; ValueSet documented | per jurisdiction |

**Supplement 2.**

**Python code like Rapidminer operators.**

This supplement provides the Python equivalent functions for the RapidMiner operators that are utilised in the article. Furthermore, scripts with an alternative implementation are also provided.

**RapidMiner operator**: Extract Statistics

**Python functions**: De las librerías pandas y Numpy

- numpy.dtype()
- pandas.DataFrame.isna.isna()
- Min() - Built-in Function
- Max() -Built-in Function
- statistics.mean()
- numpy.std()

**Example script**:

import pandas as pd

import numpy as np

import csv

from pandas.api.types import is_integer_dtype, is_float_dtype, is_numeric_dtype

def infer_type(series: pd.Series, categorical_threshold: int = 20) -> str:

    """

    Heuristic to infer variable type:

    - If numeric:

        - integer if dtype is int or all non-null values are integers

        - real otherwise

    - If not numeric: categorical

    - Optional: if unique values <= categorical_threshold, can force categorical

    """

    s = series.dropna()

    if is_numeric_dtype(series):

        if is_integer_dtype(series):

            return "integer"

        if len(s) == 0:

            return "real"

        return "integer" if np.all(np.isclose(s % 1, 0)) else "real"

    return "categorical"

def least_and_most_frequent(series: pd.Series):

    """

    Returns (least_label, least_count), (most_label, most_count)

    - Break ties alphabetically

    - NaN values are ignored

    """

    vc = series.value_counts(dropna=True)

    if vc.empty:

        return (np.nan, np.nan), (np.nan, np.nan)

    most_count = vc.max()

    least_count = vc.min()

    most_candidates = sorted([idx for idx, c in vc.items() if c == most_count], key=lambda x: str(x))

    least_candidates = sorted([idx for idx, c in vc.items() if c == least_count], key=lambda x: str(x))

    return (least_candidates[0], int(least_count)), (most_candidates[0], int(most_count))

def summarize_dataframe(df: pd.DataFrame, dtype_overrides: dict = None) -> pd.DataFrame:

    """

    Generate a summary for each column with:

    Name, Type, Missing, Minimum/Least, Maximum/Most, Average, Standard Deviation.

    Parameters:

    - df: DataFrame to summarize

    - dtype_overrides: optional dict {col: "integer"/"real"/"categorical"} to force types

    """

    rows = []

    dtype_overrides = dtype_overrides or {}

    for col in df.columns:

        s = df[col]

        col_type = dtype_overrides.get(col, infer_type(s))

        missing = int(s.isna().sum())

        min_val = max_val = avg = std = np.nan

        if col_type in ("integer", "real"):

            # Numeric: calculate min, max, mean, std

            s_num = pd.to_numeric(s, errors='coerce')

            if s_num.notna().any():

                min_val = float(np.nanmin(s_num))

                max_val = float(np.nanmax(s_num))

                avg = float(np.nanmean(s_num))

                std = float(np.nanstd(s_num, ddof=0))  # population std

        else:

            # Categorical: least and most frequent category

            (least_label, least_count), (most_label, most_count) = least_and_most_frequent(s)

            min_val = f"{least_label} ({least_count})" if pd.notna(least_label) else np.nan

            max_val = f"{most_label} ({most_count})" if pd.notna(most_label) else np.nan

        rows.append({

            "Name": col,

            "Type": col_type,

            "Missing": missing,

            "Minimum/Least": min_val,

            "Maximum/Most": max_val,

            "Average": avg,

            "Standard Deviation": std

        })

    return pd.DataFrame(rows, columns=[

        "Name", "Type", "Missing", "Minimum/Least", "Maximum/Most", "Average", "Standard Deviation"

    ])

# ===================== Example usage =====================

if __name__ == "__main__":

    # 1) Load dataset (adjust file path)

    df = pd.read_csv('CubanDataset.csv')

    # 2) Force variable types if needed

    overrides = {

        "Age": "integer",

        "Menarche": "integer",

        "Menopause": "categorical",

        "FirstBirth": "categorical",

        "BornAlive": "integer",

        "BMI": "real",

        "Alcohol": "categorical",

    }

    summary = summarize_dataframe(df, dtype_overrides=overrides)

    # 3) Print or export results

    print(summary)

    # summary.to_csv("dataset_profile_summary.csv", index=False)

    # summary.to_excel("dataset_profile_summary.xlsx", index=False)

**RapidMiner operator**: Map

**Python functions**: De las librerías pandas, pandas.DataFrame.map

**Example script 1**:

import pandas as pd

import csv

# Example CubanDataset

df = pd.read_csv('CubanDataset.csv')

# Example 1: Mapping categorical to numeric (0 = No, 1 = Yes)

df["alcohol_num"] = df["alcohol"].map({"No": 0, "Yes": 1})

# Example 2: Mapping FamilyHistory categories to number of first-degree relatives

map_family = {

    "No": 0,

    "Mother": 1,

    "Sister": 1,

    "Daughter": 1,

    "Mother/Sister": 2,

    "Mother/Daughter": 2,

    "Sister/Daughter": 2,

    "Mother/Sister/Daughter": 3

}

df["nrelbc_num"] = df["nrelbc"].map(map_family)

print(df)

**RapidMiner operator**: Impute Missing Values

**Python functions**: De la librería Pandas:

- DataFrame.fillna()
- mean()
- median()
- DataFrame.mode()

**Example script: Impute with mean() or median()**

import pandas as pd

import numpy as np

import csv

# Example CubanDataset

df = pd.read_csv('CubanDataset.csv')

print("Before imputation:")

print(df)

# Impute missing values with the mean of each column

df["imc"] = df["imc"].fillna(df["imc"].mean())

df["biopsies"] = df["biopsies"].fillna(df["biopsies"].mean())

print("\nAfter imputation with mean:")

print(df)

**Example script Impute: with mode()**

import pandas as pd

import numpy as np

import csv

# Example CubanDataset

df = pd.read_csv('CubanDataset.csv')

print("Before imputation:")

print(df)

# Impute missing values with the mode (most frequent value)

df["alcohol"] = df["alcohol"].fillna(df["alcohol"].mode()[0])

df["tobacco"] = df["tobacco"].fillna(df["tobacco"].mode()[0])

print("\nAfter imputation with mode:")

print(df)

**Example script: Impute with a constant value**:

import pandas as pd

import numpy as np

import csv

# Example CubanDataset

df = pd.read_csv('CubanDataset.csv')

print("Before imputation:")

print(df)

# Impute categorical with a constant value

df["race"] = df["race"].fillna("Unknown")

# Impute numeric with a constant value

df["exercise"] = df["exercise"].fillna(0)

print("\nAfter imputation with constant value:")

print(df)

**RapidMiner operator**: Remove Duplicates

**Python functions**: De la librería Pandas: pandas.DataFrame.drop_duplicates()

**Example script**:

import pandas as pd

import csv

# Example CubanDataset

df = pd.read_csv('CubanDataset.csv')

print("Before removing duplicates:")

print(df)

# Remove full duplicate rows

df_no_dupes = df.drop_duplicates()

print("\nAfter removing duplicates:")

print(df_no_dupes)

# --- Optional: Remove duplicates based only on specific columns ---

# Example: consider duplicates only if Age and Menarche are the same

df_no_dupes_subset = df.drop_duplicates(subset=["age", "menarche"])

print("\nAfter removing duplicates (based on age + menarche):")

print(df_no_dupes_subset)

**Supplement 3.**

**Algorithm training and performance evaluation.**

This supplement provides a generic supervised learning workflow, as depicted in Fig. 3.

**RapidMiner operators**: Naive Bayes, Generalized Linear Model (H2O), Logistic Regression (SVM), Fast Large Margin, Deep Learning (H2O), Decision Tree, Random Forest, Gradient Boosted Trees (H2O), Support Vector Machine.

**Python functions**: De las librerías Scikit-learn, statsmodel y h2o: sklearn.naive_bayes.GaussianNB(), statsmodels.glm(), sklearn.linear_model.LogisticRegression(), sklearn.svm.LinearSVC(), h2o.deeplearning(), sklearn.tree.DecisionTreeClassifier(), sklearn.ensemble.RandomForestClassifier(), sklearn.ensemble.GradientBoostingClassifier(), sklearn.svm.SVC() and sklearn.model_selection import cross_val_score().

**Example script**: Reusable skeleton for any supervised algorithm: it handles preprocessing, stratified splitting, ColumnTransformer pipeline, optional SMOTE, GridSearchCV with multiple metrics, and final reporting.

"""

Reusable supervised ML workflow adapted to /mnt/data/demo.csv

- Auto-detect dtypes; flexible preprocessing for numeric/categorical

- Stratified train/test split

- Optional SMOTE for imbalanced target

- Pipelines + GridSearchCV with multiple scorers

- Evaluate 4 models (LR, RF, SVM, GNB) and build a comparison table

Change TARGET below if your label is not 'Cancer'.

Comments are in English for MethodsX-style reproducibility.

"""

import numpy as np

import pandas as pd

from pathlib import Path

from collections import defaultdict

from sklearn.model_selection import train_test_split, StratifiedKFold, GridSearchCV

from sklearn.metrics import (

    accuracy_score, roc_auc_score, f1_score, precision_score, recall_score,

    classification_report, confusion_matrix, make_scorer

)

from sklearn.compose import ColumnTransformer

from sklearn.pipeline import Pipeline

from sklearn.preprocessing import OneHotEncoder, StandardScaler

from sklearn.impute import SimpleImputer

# Optional (class imbalance handling)

try:

    from imblearn.over_sampling import SMOTE

    from imblearn.pipeline import Pipeline as ImbPipeline

    HAS_IMBLEARN = True

except Exception:

    HAS_IMBLEARN = False

# ------------------------ 1) Load data ------------------------

CSV_PATH = Path("CubanDataset.csv")  # adjust if needed

TARGET = "cancer"                      # change this if your target column has a different name

df = pd.read_csv(CSV_PATH)

# Safety checks and basic cleaning for target

if TARGET not in df.columns:

    raise ValueError(f"Target column '{TARGET}' not found in {CSV_PATH.name}. "

                     f"Available columns: {list(df.columns)}")

# Map Yes/No textual labels to 1/0 if needed

if df[TARGET].dtype == object:

    df[TARGET] = df[TARGET].str.strip().str.title().map({"Yes": 1, "No": 0})

# Ensure binary integer target

df[TARGET] = pd.to_numeric(df[TARGET], errors="coerce")

if df[TARGET].dropna().nunique() != 2:

    raise ValueError(f"Target '{TARGET}' must be binary. Found unique values: {df[TARGET].dropna().unique()}")

df = df.dropna(subset=[TARGET])  # drop rows with missing target

y = df[TARGET].astype(int)

X = df.drop(columns=[TARGET]).copy()

# ------------------------ 2) Identify column types ------------------------

# Numeric = float/int; Categorical = object or boolean

# If you have integer-coded categories (e.g., Race=1/2/3), they will be treated as numeric here.

numeric_features = [c for c in X.columns if pd.api.types.is_numeric_dtype(X[c])]

categorical_features = [c for c in X.columns if (X[c].dtype == "object") or (pd.api.types.is_bool_dtype(X[c]))]

# If there are no categorical features, keep list empty

# If there are no numeric features, keep list empty

# (ColumnTransformer will handle either case.)

# ------------------------ 3) Preprocessing blocks ------------------------

numeric_transformer = Pipeline(steps=[

    ("imputer", SimpleImputer(strategy="median")),     # robust to skew/outliers

    ("scaler", StandardScaler(with_mean=True, with_std=True))

])

categorical_transformer = Pipeline(steps=[

    ("imputer", SimpleImputer(strategy="most_frequent")),

    ("ohe", OneHotEncoder(handle_unknown="ignore", sparse_output=False))

])

preprocess = ColumnTransformer(

    transformers=[

        ("num", numeric_transformer, numeric_features),

        ("cat", categorical_transformer, categorical_features)

    ],

    remainder="drop"

)

# ------------------------ 4) Models & grids ------------------------

from sklearn.linear_model import LogisticRegression

from sklearn.ensemble import RandomForestClassifier

from sklearn.svm import SVC

from sklearn.naive_bayes import GaussianNB

models_and_grids = {

    "LogisticRegression": {

        "estimator": LogisticRegression(max_iter=1000, class_weight="balanced"),

        "param_grid": {

            "clf__C": [0.01, 0.1, 1, 10],

            "clf__solver": ["lbfgs", "liblinear"],

            "clf__penalty": ["l2"]

        }

    },

    "RandomForest": {

        "estimator": RandomForestClassifier(class_weight="balanced", random_state=42),

        "param_grid": {

            "clf__n_estimators": [200, 400],

            "clf__max_depth": [None, 8, 16],

            "clf__min_samples_split": [2, 10],

            "clf__min_samples_leaf": [1, 3]

        }

    },

    "SVM": {

        "estimator": SVC(probability=True, class_weight="balanced", random_state=42),

        "param_grid": {

            "clf__C": [0.1, 1, 10],

            "clf__kernel": ["rbf", "linear"],

            "clf__gamma": ["scale", "auto"]

        }

    },

    "GaussianNB": {

        "estimator": GaussianNB(),

        "param_grid": {

            # Optional smoothing search:

            # "clf__var_smoothing": np.logspace(-12, -6, 7)

        }

    }

}

# ------------------------ 5) CV & scoring ------------------------

cv = StratifiedKFold(n_splits=5, shuffle=True, random_state=42)

scorers = {

    "accuracy": make_scorer(accuracy_score),

    "roc_auc": make_scorer(roc_auc_score, needs_proba=True),

    "f1": make_scorer(f1_score),

    "precision": make_scorer(precision_score, zero_division=0),

    "recall": make_scorer(recall_score)

}

# ------------------------ 6) Train/test split ------------------------

X_train, X_test, y_train, y_test = train_test_split(

    X, y, test_size=0.25, stratify=y, random_state=42

)

# ------------------------ 7) Fit each model via GridSearchCV ------------------------

USE_SMOTE = False and HAS_IMBLEARN  # set to True if imbalanced and imblearn is available

results = []

best_estimators = {}

for name, cfg in models_and_grids.items():

    estimator = cfg["estimator"]

    param_grid = cfg["param_grid"]

    if USE_SMOTE and HAS_IMBLEARN:

        pipe = ImbPipeline(steps=[

            ("preprocess", preprocess),

            ("smote", SMOTE(random_state=42)),

            ("clf", estimator)

        ])

        # You could also grid over SMOTE params:

        # param_grid = {**param_grid, "smote__k_neighbors": [3, 5]}

    else:

        pipe = Pipeline(steps=[

            ("preprocess", preprocess),

            ("clf", estimator)

        ])

    grid = GridSearchCV(

        estimator=pipe,

        param_grid=param_grid,

        scoring=scorers,  # multiple scorers

        refit="roc_auc",  # primary metric

        cv=cv,

        n_jobs=-1,

        verbose=0

    )

    grid.fit(X_train, y_train)

    best_estimators[name] = grid.best_estimator_

    # Cross-validated scores (best params)

    cv_scores = grid.cv_results_

    best_idx = grid.best_index_

    cv_summary = {m: cv_scores[f"mean_test_{m}"][best_idx] for m in scorers.keys()}

    # Test set evaluation

    model = grid.best_estimator_

    y_pred = model.predict(X_test)

    y_proba = model.predict_proba(X_test)[:, 1] if hasattr(model, "predict_proba") else None

    test_metrics = {

        "test_accuracy": accuracy_score(y_test, y_pred),

        "test_f1": f1_score(y_test, y_pred),

        "test_precision": precision_score(y_test, y_pred, zero_division=0),

        "test_recall": recall_score(y_test, y_pred),

        "test_roc_auc": roc_auc_score(y_test, y_proba) if y_proba is not None else np.nan

    }

    # Store row

    row = {

        "Model": name,

        "BestParams": grid.best_params_,

        "CV_mean_accuracy": cv_summary["accuracy"],

        "CV_mean_roc_auc": cv_summary["roc_auc"],

        "CV_mean_f1": cv_summary["f1"],

        "CV_mean_precision": cv_summary["precision"],

        "CV_mean_recall": cv_summary["recall"],

        **test_metrics

    }

    results.append(row)

    # Optional: print quick report

    print(f"\n=== {name} ===")

    print("Best params:", grid.best_params_)

    print("CV (refit metric) best score:", grid.best_score_)

    print("Test metrics:", {k: round(v, 3) if pd.notna(v) else v for k, v in test_metrics.items()})

    print("Confusion matrix:\n", confusion_matrix(y_test, y_pred))

    print("Classification report:\n", classification_report(y_test, y_pred, digits=3, zero_division=0))

# ------------------------ 8) Comparison table ------------------------

comparison = pd.DataFrame(results).sort_values(by="test_roc_auc", ascending=False)

print("\n=== Model comparison (sorted by test ROC AUC) ===")

print(comparison)

# Optional: save results

# comparison.to_csv("model_comparison_demo.csv", index=False)

**How to use it**:

- Enable SMOTE by setting USE_SMOTE = True if there is an imbalance and you have imblearn.
- Change refit="roc_auc" to your primary metric (e.g., "accuracy").
- Change the classifier and its grid_param to try a different algorithm:
  - Naive Bayes: sklearn.naive_bayes.GaussianNB(),
  - Generalized Linear Model: sklearn.linear_model.TweedieRegressor(),
  - Logistic Regression: sklearn.linear_model.LogisticRegression(),
  - Fast Large Margin (FLM): sklearn.svm.LinearSVC(),
  - Deep Learning: sklearn.neural_network.MLPClassifier() o h2o.deeplearning(),
  - Decision Tree: sklearn.tree.DecisionTreeClassifier(),
  - Random Forest: sklearn.ensemble.RandomForestClassifier(),
  - Gradient Boosted Trees: sklearn.ensemble.GradientBoostingClassifier(),
  - Support Vector Machine: sklearn.svm.SVC()
